# Sleep position, patient comfort, and technical performance with two established procedures for home sleep testing

**DOI:** 10.1007/s11325-021-02530-w

**Published:** 2021-12-31

**Authors:** C. Emika Mueller, Hansen Li, Sophia M. Begasse, J. Ulrich Sommer, Boris A. Stuck, Richard Birk

**Affiliations:** 1 Department of Otorhinolaryngology, University Hospital Marburg, Philipps-Universität Marburg, Baldingerstraße, 35043 Marburg, Germany; 2 Department of Otorhinolaryngology, University Hospital Munich, Technische Universität München, München, Germany

**Keywords:** Positional sleep apnea, Sleep position, Obstructive sleep apnea, Sleep-disordered breathing, Polygraphy, Peripheral arterial tonometry

## Abstract

**Purpose:**

In patients with a high pre-test probability of suffering from obstructive sleep apnea (OSA), (cardio)-respiratory polygraphy (RP; level 3) is commonly used for home sleep testing (HST); however, testing based on peripheral arterial tonometry (PAT) is increasingly recognized as an alternative method. The aim of the study was to compare sleep position, patients’ comfort, and technical failure rates of HST with RP and PAT in patients with suspected OSA.

**Methods:**

Sleep position, patients’ comfort, and technical failure rates of RP and PAT were compared in 56 patients receiving two nights of HST with either RP or PAT in a randomized fashion.

**Results:**

Time in supine position with PAT was significantly lower (173.7±88 min) compared to RP (181.7±103.7 min; *p* < 0.001), although the absolute mean difference was not clinically significant. Patients reported to sleep better, feeling less disturbed when falling asleep, losing less sensors, and fewer nightly awakenings with PAT, but experienced more pain at the side of the finger probe. Forty-five out of 56 patients (80%) rated PAT as being the superior sleep test and 49 out of 56 (88%) would prefer PAT for further investigations (*p*<0.001). PAT testing was associated with less technical failures.

**Conclusion:**

The results demonstrate that HST with PAT leads to less time in supine sleep positioning, which may be clinically relevant in selected patients. Moreover, PAT is associated with less technical failures and is perceived with less discomfort during testing and a reduced number of nocturnal awakenings in patient self-reports.

## Introduction

Since the mid-1980s, sleep apnea has been recognized as an independent risk factor for cardio- and cerebrovascular, endocrine, and psychiatric diseases [1–3]. Obstructive sleep apnea (OSA) has high and increasing prevalence of 9–38% [4], especially among patients with specific risk factors such as male sex or obesity [5–8]. Reduced tension of the pharyngeal muscles during sleep leads to obstruction of the upper airway, leading to respiratory events of apnea and hypopnea, resulting in repetitive arousals. These nocturnal arousals result in daytime sleepiness, reduced performance, and an impairment in sleep-related quality of life [9].

Sleep position is a major contributing factor where the supine position is vulnerable for apnea events [10]. In the literature, positional OSA (pOSA) is most commonly defined as a supine to non-supine ratio in the apnea-hypopnea index (AHI) of ≥2 [10, 11]. The prevalence of pOSA has been estimated to be 25 to 30% and particularly a factor among patients with milder OSA and lower body mass index [10, 12]. Patients with supine isolated OSA (siOSA) show respiratory disturbances exclusively in supine position and otherwise a normal AHI and positional therapy to prevent the supine position has high therapeutic potential [13]. Therefore, sleep testing should include the possibility to objectify sleep position. Level 3 home sleep tests (HST), such as (cardio)-respiratory polygraphy (RP), are well validated and can be used to diagnose OSA among patients with a high pre-test probability determined through sleep medical history and clinical examination [14–16]. However, testing based on peripheral arterial tonometry (PAT) is increasingly recognized as an alternative method [16–20]. The PAT method consists of a smart-watch-sized computer and two sensors (one worn on the finger and another worn on the chest). Compared to RP, PAT has less technical equipment being attached to the sleeping patient. This may lead to a more natural sleeping behavior with less supine body position, a higher patient comfort, and less recording failures during sleep. It is demonstrated by Vonk et al. that polysomnography (PSG) apparatus leads to more supine position during sleep, causing an overestimation of OSA severity, especially in patients with pOSA [21]. To date, a comparison between PSG similar RP systems and PAT HST has not been performed. With this regard, we performed a randomized controlled study with two nights of testing with either RP or PAT to test the hypothesis that PAT-based HST with its reduced technical equipment is superior to RP regarding its (non)-influence on sleep position (primary outcome), patient comfort, and technical failure rates (secondary outcome).

## Methods

This prospective study was conducted from January to July 2020 at a tertiary referral center (university clinic) and approved by the local ethics committee (study number 105/19). The study was designed and performed in accordance to the Good Clinical Practice guidelines and the Declaration of Helsinki (EN ISO 14155). The participants were recruited during the outpatient sleep clinic and participation was offered to all adult patients needing HST for suspected sleep-disordered breathing, in terms of loud irregular snoring, witnessed apneas, and daytime sleepiness. All participants agreed to the study protocol and written informed consent was obtained. Exclusion criteria included physical or mental restrictions interfering with independently installing the devices. In addition, patients with musculoskeletal diseases were not included. All participants received a RP- and a PAT-based HST device in a randomized order in two consecutive nights. The randomization list was generated using Microsoft Excel (Version 16.0) and given to the research assistant in closed envelopes. After recruitment and education by the physician, the patient was instructed in the devices by the research assistant, the randomization envelope was opened, and the patient was informed regarding the sequence. Patient management, device distribution, and education were provided by the same research assistant during the entire study.

As RP reports respiratory events in relation to recording time instead of total sleep time, it is suggested to use the term “respiratory event index” instead of AHI. As PAT reports respiratory events in relation to total sleep time, AHI is the correct terminology. For better readability however, AHI is used for both methods of HST throughout the manuscript. According to the International Classification of Sleep Disorders, OSA was defined as an AHI ≥ 5/h in combination with symptoms or comorbidities [22]. Mild OSA was defined as an AHI of 5 to <15, moderate OSA was defined as an AHI of 15 to <30, and severe OSA was defined as an AHI ≥ 30.

### Nocturnal testing and technical failure definition

RP was conducted with the Miniscreen plus (Heinen und Löwenstein, Bad Ems, Germany) and consisted of the following channels: Dynamic ventilation pressure sensor to detect respiratory airflow, abdominal and chest straps with a pressure sensor to detect thoracic and abdominal movements (breathing effort), pulse oximetry for recording oxygen saturation (SpO_2)_ and heart rate, microphone to detect snoring, position sensor to detect the body position and light sensor. The recording was scored manually with the corresponding software (Version 5.19) according to the 2012 update of the procedures and definitions from the American Academy of Sleep Medicine (AASM) as published in 2007 [23]. Hypopnea was defined as a drop in peripheral oxygen saturation of 3% and a reduction in respiratory flow between 30 and 90% for at least 10 s compared to the baseline. Technically adequate testing was defined as all channels providing non-interrupted information for at least 6 h. If this was not the case, the testing was scored as a technical failure with the need for repeated testing.

PAT-based examination was conducted with WatchPAT200/300 (Itamar Medical Ltd., Caesarea, Israel) which works without conventional sensors such as airflow and breathing effort [24]. It consists of the following channels: a pressurized finger probe to detect PAT signal, oxygen saturation (SpO_2)_ and heart rate, a wrist sensor for actigraphy and a chest sensor for body position, snoring sound analysis and chest motion. The examination was manually edited with the zzzPAT Software (Version 5.1.76.3) according to the manufacturers’ scoring guidelines based on the “Comparison of WatchPAT with Sleep Studies (COMPASS)” project. Apneas and hypopneas are identified by presence of sympathetic activation, characterized by a typical “reciprocal” pattern of PAT amplitude reduction coinciding with an increase in heart rate. Scoring is then based on the combination of these reciprocal patterns with oxygen saturation, snoring sounds and sleep stage as described in positive validation studies [25, 26]. The same criteria for a technically successful testing were established as for the RP.

### Patients’ discomfort and satisfaction

Participants completed a questionnaire to assess testing discomfort and device preference (see Table 1).

**Table 1:** Questionnaire and answers evaluating the quality of sleep and discomfort levels for participants using respiratory polygraphy (RP) and peripheral arterial tonometry (PAT)

	**RP**				**PAT**		
**Question**	**Yes**	**No**	/*		**Yes**	**No**	/*
1. Did you sleep well tonight?	20 (39%)	32 (61%)	4		39 (74%)	15 (26%)	3
2. Was the examination disturbing when you fell asleep?	33 (60%)	22 (40%)	1		6 (12%)	46 (78%)	4
3. Did you lose any sensors (nasal cannula, finger sensor, belts) during the night?	11 (20%)	44 (80%)	1		3 (6%)	51 (94%)	2
4. Was the examination painful?	3 (5%)	52 (95%)	1		7 (13%)	47 (87%)	2
5. Did the examination wake you up?	28 (50%)	27 (50%)	1		16 (30%)	38 (70%)	2
Number of wake-ups:	Mean 1.8; Median 1				Mean 0.62; Median 0		
6. How do you rate the overall sleeping comfort with the test device?	Visual scale with thumbs up and thumbs down in 5 steps (grade 1 (best) to 5 (worse); see Fig. 2.)						
After both nights of testing they were finally asked:							
7. Which system did you sleep better with?	11 (20%)			45 (80%)			
8. Which system would you prefer for future testing?	6 (12%)			49 (88%)			

^/*^ = Number of patients who abstained from the questions. % is related to the total number of patients who have answered the questions

### Statistical analysis

RP- and PAT-based position data and rating/preference were statistically analyzed using the Wilcoxon-Mann-Whitney test, as the Kolmogorov Smirnoff test showed a deviation on normal distribution. Statistical differences were analyzed using Fisher’s test with the repeated investigations. Statistical analyses and plotting were performed using R, an open source environment for statistical computing and graphics [27].

## Results

### Demographic parameters

Sixty-one patients were included in the study. Five patients were excluded from the analysis, as only one test was performed due to logistics and loss to follow-up. This included one complete testing failure with the RP due to a miscommunication with the technical staff, one operating error by the patient with PAT system, and one early termination of the PAT system due to pain from the finger probe in a patient with unusually large fingers.

In the remaining cohort of 56 patients, age ranged from 19 to 76 years (mean age: 44 ± 12 years and median age: 44 years) with a BMI ranging from 19.2 to 41.4 kg/m^2^ (median BMI 27.3 kg/m^2^, mean value 28.1 kg/m^2)^. Thirty-nine (68%) of the participants were male. Figure 3 is showing the patient flow.

### OSA and position analysis

OSA was diagnosed with PAT and/or RP in 51 cases. With RP, OSA was diagnosed in 41 and with PAT in 50 cases. Average AHI with PAT testing was 23.9 ± 17.6/h and 17.5 ± 14/h with RP. One patient, who was not diagnosed with OSA using PAT, was diagnosed with mild non-positional OSA (npOSA) with RP. Nine patients were diagnosed with OSA with PAT, but did not have abnormal scores with RP (4 with mild pOSA, 3 with mild npOSA, 1 with intermediate npOSA, 1 with severe npOSA).

Mild OSA was diagnosed with RP in 22 cases and with PAT in 19 cases, intermediate OSA in 13/17 and severe OSA in 7/14 cases. Details are shown in Table 2.

**Table 2:** Apnea-hypopnea index (AHI) in respiratory polygraphy (RP) and peripheral arterial tonometry (PAT) examinations in patients with obstructive sleep apnea (OSA)

	AHI ≥ 5/h	Mild OSA	Moderate OSA	Severe OSA
RP	41	22	13	6
PAT	50	19	17	14
PAT or RP	51			

Mild: 5/h≤ AHI <15/h. Intermediate: 15/h≤ AHI < 30/h. Severe: AHI ≥ 30/h

More patients with positional and supine isolated OSA were diagnosed with PAT than with RP. Diagnosed cases of pOSA and siOSA with PAT or RP are shown in Table 3.

**Table 3:** Apnea-hypopnea index (AHI) in respiratory polygraphy (RP) and peripheral arterial tonometry (PAT) examinations in patients with obstructive sleep apnea (OSA)

PAT *n*=51	AHI ≥ 5/h	Mild OSA	Moderate OSA	Severe OSA
pOSA	15	4	5	6
siOSA	7	7	0	0
Total	22	11	5	6
RP *n*= 46	AHI ≥ 5/h	Mild OSA	Moderate OSA	Severe OSA
pOSA	8	2	6	0
siOSA	5	4	1	0
Total	13	6	7	0

Mild: 5/h≤ AHI <15/h. Intermediate: 15/h≤ AHI < 30/h. Severe: AHI ≥ 30/h. *pOSA* positional OSA, *siOSA* supine isolated OSA pOSA and non-supine AHI < 5

**Fig. 1:**
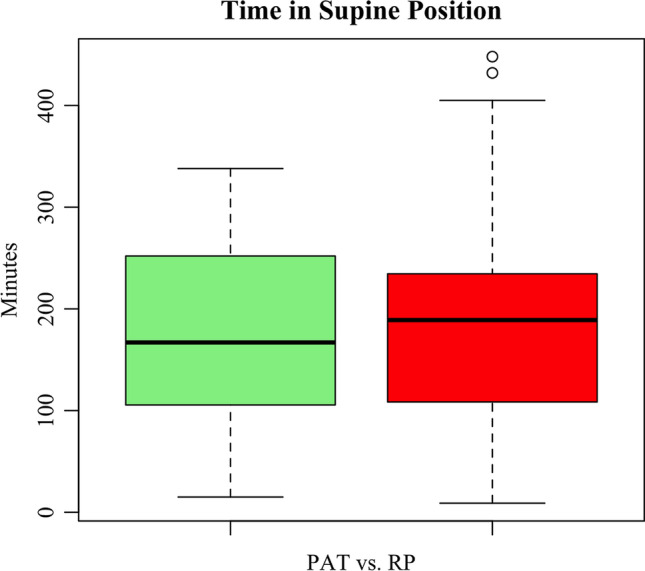
Box and whisker plot of time (minutes) spent in supine position with respiratory polygraphy (RP)– and peripheral arterial tonometry (PAT)–based examinations. The boxes represent the interquartile range (IQR) with the whiskers extending up to 1.5 times the IQR. The median is marked with a solid black line. Outliers are marked with a circle. Green box = PAT. Red box = RP

Time in supine position with PAT was significantly lower (173.7 ± 88 min; median: 167 min) compared to the time in supine position with RP (181.7 ± 103.7 min; median: 189 min; *p* < 0.001). Details are shown in Fig. 1. Regarding a possible order effect, there is no significant difference between the first (193.6 min ± 99.9 min; median: 186 min) and the second night (161.8 ± 91.5 min; median: 156, *p* = 0.4) independent of the system used.

**Fig. 2:**
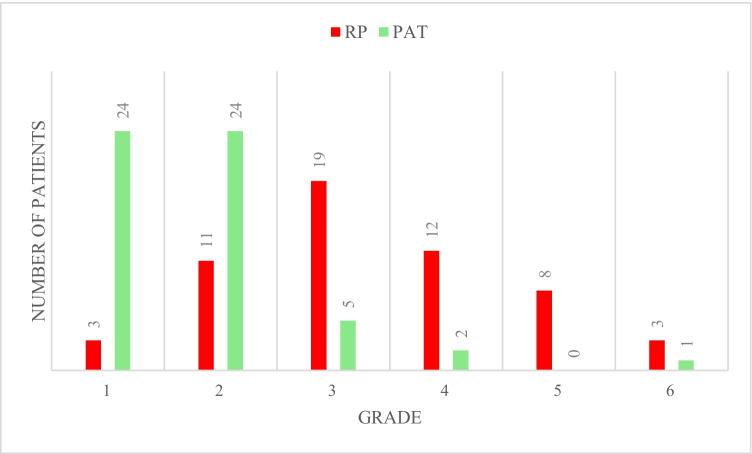
Overall sleeping comfort in respiratory polygraphy (RP)- and peripheral arterial tonometry (PAT)–based examinations. Self-reported sleeping comfort grades: 1 = best, 5 = worst. 6 = no rating reported. Red = RP. Green = PAT

Individual effects on body position for each participant are presented in a waterfall plot (Fig. 2).

### Patients’ discomfort and satisfaction

Among 55 patients completing the questionnaire following RP testing, 20/52 (39%) participants stated that they slept well during the night and 33/55 (60%) answered that the RP was disturbing when they went to sleep. Eleven out of 55 patients (20%) stated that they lost sensors during the night. Three out of 55 (5%) experienced pain due to the nasal cannula dynamic pressure measurement and due to the device itself. Awakenings subjectively related to the testing device were reported in 28/55 (50%) cases. The number of awakenings ranged from 0 to 10 times with a mean of 1.8 and a median of 1.

Among 54 patients completing the questionnaire following PAT testing, 39/54 (74%) of the patients stated that they slept well during the night with PAT. Six out of 53 (12%) patients answered that the PAT was disturbing when they went to sleep. Three out of 54 (6%) patients lost sensors during the night with PAT. Seven out of 54 (13%) experienced pain in the exposed finger during PAT. Sixteen out of 54 (30%) of the patients reported to have woken up during PAT testing. This subjective perceived number of awakenings ranged from 0 to 6 times with PAT. On average, patients woke up 0.62 times with PAT with a median of 0 times.

In comparison, patients slept better during the night, felt less disturbed when falling asleep, suffered less sensor loss during the night and reported less nightly awakening with the PAT testing. In contrast, more patients experienced pain (at the side of the finger probe) during the night with PAT. The subjective perceived number of awaking was significantly lower during PAT compared to RP (*p*=0.004). Details of the first part of the questionnaire for RP and PAT are shown in Table 1.

**Fig. 3:**
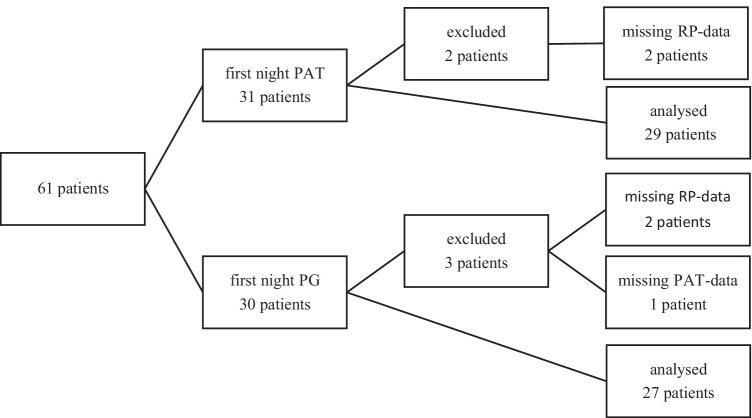
Patient flow during the study

Details of the question number 6, “How do you rate the overall sleeping comfort with the system?” are shown in Fig. 3 for RP and PAT.

Significantly more patients 45/56 (80%) rated PAT as being the superior sleep test and significantly more patients 49/56 (88%) would prefer PAT for further investigations (*p*<0.001).

### Necessity of repeating the examinations

Regarding the entire cohort of 61 subjects, PAT testing was repeated twice due to an operating error, as two patients forgot to switch on the device. Technical failures related to insufficient recording time or loss of sensors did not occur.

RP needed to be repeated 8 times in different patients. In two patients, the recording time of the RP was under 6 h (1:46h/5:15h) due to unknown technical failures. Two additional times the device did not start recording due to an operating error by the technical assistant. Finally, additional 3 patients required repeating PR studies due to loss of the nasal pressure sensor or inadequate examination time (< 4 h of examination achieved). There are no statistical differences in the necessity of repeating the examinations (*p* =0.22) but there is a tendency that the PAT testing is associated with less failures. It is necessary to mention that the PAT examination was not possible in one patient due to size restriction in the finger sensor; this patient was excluded from the study.

## Discussion

Reliable testing is necessary to address the high prevalence and the medical consequences of OSA. Objective identification of body position is increasingly relevant in OSA management, e.g., with positional therapy (PT) as a standalone therapy or in combination with, e.g., mandibular advancement devices (MAD) [28–30].

Here, we compared two established HST systems in regarding their identification of sleep position, patients’ comfort, overall satisfaction and technical failure rates. More patients were diagnosed with OSA with PAT compared to RP and the mean AHI was higher with PAT testing. This may be related to the fact that RP reports AHI in relation to recording time (as sleep staging is not assessed) while PAT reports AHI in relation to total sleep time. Therefore, OSA severity may be underestimated by RP when AHI is “diluted” due to longer recording times that include sleep and wakefulness. However, it is known that AHI may be underestimated in RP compared to PSG especially if pre-test probability for OSA is low or in children [31, 32]. On the other hand, RP is a validated method to diagnose OSA with high pre-test probability [15]. Another point making PAT more sensitive in the testing of OSA is the scoring of respiratory events (reciprocal pattern and snoring or desaturation). This might be comparable to the scoring of a respiratory effort–related arousals (RERAs) in PSG. Since the PAT-RDI correlates well with the RDI of the PSG, the system may be more sensitive compared to RP [33].

When interpreting our results, it must be taken into account that several studies have shown that there are considerable differences in AHI in different (consecutive) nights [34–36], even if there is a recent study in 99 patients with OSA demonstrating that the night-to-night variability in the AHI and the sleep time in supine position over three consecutive nights were not statistically significant during a level 3 HST [34].

The time spent in supine position with PAT was significantly lower than with the RP. This effect may reflect what has been described in other studies with PSG testing. It may be related to the reduced technical equipment of the PAT testing, especially the lack of a chest and abdominal belt and the device itself [21]. This may be particularly relevant in pregnant women, as previously described [37]. In 1985, Cartwright addressed in a study that more research needs to be done because of patients feeling constrained by monitors and sensors so they would sleep more in supine position as they would normally do at home [38]. Metersky showed effects of PSG on sleep position within 12 participants that supine position with PSG equipment was 49%, which was 56% higher than without PSG equipment [39].

In 2018, Wimaleswaran showed in a study of 19 participants with 3 nights at home and 3 nights inhouse PSG that supine position at home (measured only with a sleep position sensor) was lower in 13 participants. The overall supine position was 35% with PSG and 25% at home. He stated that this finding could “potentially modify treatment recommendations” [40]. The latest retrospective study about the effect of in-lab PSG and HST with PAT on sleep position showed that supine position in 445 PSG vs. 416 PAT did not differ [41]. However, the study has its limitations, as the different devices were not compared within the same patient. To date, there is no comparison in the literature between RP and PAT testing regarding the sleep positions. Although the absolute difference in the time spent in the supine position for the entire cohort is relatively small, this may lead to clinically relevant effects (in both directions) in selected patients as demonstrated in the individual comparison.

In this study, patients reported feeling generally less disturbed during the night with the PAT compared to RP, as reflected by the results of the questionnaires. As an overall assessment, 88% of the participants would prefer PAT testing. After showing PAT efficacy, reliability, and reproducibility in 102 participants (69 with OSA) for diagnosing OSA with the WatchPAT^®^ 100 device compared to in-laboratory, manual-scored standard PSG by Bar et al. in 2003, many studies have validated the PAT technology in the diagnosis of OSA also in adolescents and during pregnancy [17, 18, 37, 42].

A relevant limitation for the PAT system is discomfort at the side of the finger probe. The diagnostic algorithm for the PAT system is validated for the index finger, although clinical experience demonstrates that the index fingers of some patients are too big for the probe, which is only available in one size. Further research and technical developments are required to validate the system for non-index fingers or regarding the availability of different sizes of the finger probe.

The PAT-based testing needed to be repeated in two patients due to failing to switch on the device at the beginning of the night. The RP needed to be repeated 8 times (once in eight patients), because of insufficient signals (a loss of oxygen sensor or the nasal dynamic pressure sensor). With this regard, PAT seems to be the more robust testing method. Further improvements may be achieved by providing the option to define a fix start of the recording by the investigator as long as the finger probe is in place.

Besides the limitations mentioned above, our study has limitations as the sample size was reduced to 56 patients. However, the randomized design allows a direct comparison between the two systems.

## Conclusion

These data demonstrate that HST with the PAT system might lead to less time in supine position which may be clinically relevant in selected patients. Moreover, PAT is associated with less patients’ discomfort during testing, a reduced number of nocturnal awakenings in subjective assessment, and technically more robust test results with a reduced number of re-testing due to technical failure.
